# Training set designs for prediction of yield and moisture of maize test cross hybrids with unreplicated trials

**DOI:** 10.3389/fpls.2023.1080087

**Published:** 2023-03-06

**Authors:** Jérôme Terraillon, Frank K. Roeber, Christian Flachenecker, Matthias Frisch

**Affiliations:** ^1^ Institute of Agronomy and Plant Breeding II, Justus Liebig University, Giessen, Germany; ^2^ Corteva AgriScience, Munchen, Germany; ^3^ Eckernförde, Germany

**Keywords:** unreplicated trials, training set, maize, cross validation, genomic prediction

## Abstract

Unreplicated field trials and genomic prediction are both used to enhance the efficiency in early selection stages of a hybrid maize breeding program. No results are available on the optimal experimental design when combining both approaches. Our objectives were to investigate the effect of the training set design on the accuracy of genomic prediction in unreplicated maize test crosses. We carried out a cross validation study on basis of an experimental data set consisting of 1436 hybrids evaluated for yield and moisture for which genotyping information of 461 SNP markers were available. Training set designs of different size, implementing within environment prediction, within year prediction, across year prediction, and combinations of data sources across years and environments were compared with respect to their prediction accuracy. Across year prediction did not reach prediction accuracies that are useful for genomic selection. Within year prediction across environments provided useful correlations between observed and predicted breeding values. The prediction accuracies did not improve when adding to the training set data from previous years. We conclude that using all data available from unreplicated tests of the current breeding cycle provides a good accuracy of predicting test crosses, whereas adding data from previous breeding cycles, in which the genotypes are less related to the tested material, has only limited value for increasing the prediction accuracy.

## 1 Introduction

Genomic prediction of the performance of selection candidates has the potential to increase the selection intensity and the selection gain in breeding programs (cf de los Campos et al., 2013; Wang et al., 2018; Fritsche-Neto et al., 2021).

Choosing a suitable training set to estimate genetic effects affects the accuracy of the genomic predictions. With simulations Hickey et al. (2014) derived guidelines for the design of the training set used to predict the performance of genotypes derived from biparental populations. In maize, Technow et al. (2012) investigated the use of combined vs. separated training set for the heterotic groups. Incorporating dominance effects and using population specific marker effects in the prediction model was investigated by Technow et al. (2013). Lorenz and Smith (2015) concluded that in barley the accuracy of genomic prediction benefits from focusing on small training sets closely related to the prediction set. Brauner et al. (2020) found that using unrelated genotypes in the training set can even have negative effects on prediction accuracy. The efficiency of genomic selection in highly structured populations was investigated by Rincent et al. (2012). Training set composition in maize breeding programs with repeated field trials was investigated by Albrecht et al. (2011) and Schrag et al. (2019). Genomic prediction of single crosses in multiple environments in early stages of a maize breeding program was investigated by Massman et al. (2013).

Integrating genomic prediction in breeding programs and at the same time reducing the expenses for field trials was the subject of recent studies. Jarquin et al. (2020) conducted genomic prediction with reduced multi-environment breeding trials in which not all genotypes of interest are grown in each environment. Verges and Van Sanford (2020) investigated genomic prediction with wheat lines evaluated in a few environments with one plot per environment. Across year prediction was used to add field trial data from previous years to the training set for predicting the genotypes of the current year (Wang et al., 2020). This approach increased the prediction accuracy while limiting the amount of trials in the actual year. Training set optimization with historical data was investigated in wheat (Sarinelli et al., 2019). However common genotypes have to be tested each year to ensure a good prediction accuracy (Auinger et al., 2016).

Unreplicated trials can be used in early stages of a breeding program, when either the amount of seeds available is limited or when a given number of selection candidates have to be tested with limited resources. In unreplicated trials, the effect of the genotype is confounded with the genotype by environment interaction effect. This confounding reduces the efficiency of selection. However, a prerequisite of a successful variety is, that its performance is high across environments. Therefore a superior genotype should be among the best selection candidates in any of the evaluation environments. In addition to those genotypes that show a high performance due to their high genotypic value, genotypes that perform well due to a favorable genotype by environment interaction or a large experimental error are selected. The latter genotypes will be selected out in later stages of the breeding program due to their weaker performance in the additional environments.

Application of unreplicated trials was suggested in cereal breeding programs by Endelman (2014) and the first results are promising (Michel et al., 2017; Michel et al., 2019; Terraillon et al., 2022). To our knowledge, no concepts are available for implementing genomic pre-selection in a maize breeding program that uses unreplicated test crosses.

Our goal was to use a data set of 1436 test cross hybrids evaluated in unreplicated field trials for yield and moisture in a cross validation study to investigate the design of the training set for genomic prediction. In particular, our objectives were to compare training set designs implementing (a) within environment prediction, (b) within year prediction, (c) across year prediction, and (d) combinations of data sources across years and environments with respect to their prediction accuracy.

## 2 Material and methods

### 2.1 Genetic material, phenotyping and genotyping

The genetic material used for this study consisted of 1436 Dent x Flint test cross hybrids from a commercial breeding program. The parental components were doubled haploid lines. The hybrids were evaluated in an unreplicated trial in years 2013, 2014, and 2015 at seven locations for yield and moisture. We use the term unreplicated trial in this study for a trial, in which a selection candidate was evaluated in one single plot at one environment. No control or standard genotypes were used across trials. The number of hybrids tested in an environment ranged from 89 to 110 (**Table 1**).

**Table 1:** Number and type of hybrids tested at locations 1 to 7 in years 2013 to 2015.

Year	LOC1	LOC2	LOC3	LOC4	LOC5	LOC6	LOC7
2013	DT1 x 103	DT6 x 109 DT11 x 1	DT7 x 89		FT4 x 96	FT5 x 94	FT11 x 94
						
2014	FT2 x 106	FT12 x 106	FT9 x 106	FT10 x 106			
2015	DT13 x 106	DT3 x 10				FT9 x 88FT12 x 19	FT10 x 75FT12 x 32
					

For each set of hybrids the tester as well as the number of test cross candidates is given. FT is a Flint tester and DT is a Dent tester. For example, in year 2013 at location 1, the Dent tester DT1 was crossed with 103 Flint test cross candidates.

Markers have been obtained by a custom Illumina chip. The number of 481 SNPs markers were assessed for the 726 parental lines of the 1436 hybrids. We preprocessed the data so that all SNPs had exactly two variants, a minimum expected heterozygosity ( $1-f_{1}^{2}-f_{2}^{2}$ , where *f*_1_ and *f*_2_ are frequencies of the two SNP variants in the set of parental lines) of 10%, and were available for at least 90% of the parental lines. The frequency of missing SNPs in a parental line did not exceed 10%. The preprocessing of the data reduced the number of SNPs to 461. The distribution of the markers on the chromosomes as well as their average and maximum distance per chromosome are presented in the **Table 1** of the supplementary material (**Supplementary Table S1**). These markers allow the distinction between the Flint and Dent pools as shown by a PCA and a heatmap analysis of the lines based on the SNPs (**Supplementary Figures S1**, **S2**). Additionally, the LD decay was investigated for both pools (**Supplementary Figures S3**, **S4**). Pairwise genetic distances between hybrids in one environment, and distances between all pairs of hybrids in two environments have been calculated based on the SNPs markers using the Roger’s distance. A table presenting these results is available in the supplementary material (**Supplementary Table S2**). Corteva AgriScience owns the tested germplasm and has conducted the field experiments and marker analysis.

### 2.2 Phenotypic values adjusted for environmental effects

In a first analysis step, we adjusted the phenotypic data for environmental effects (for convenience we omit indices and present the formulas for scalar values). We estimated with analysis of variance methodology the year effects *q* and the environmental effects *p*(*q*) from the fixed effects model


(1) y=μ+q+p(q)+e

where *y* are the unreplicated plot values, *μ* is the general mean and *e* is the experimental error. Then we calculated adjusted plot values


(2) $$y^{*}=y-q-p\left(q\right).$$

The estimated effects for years and environments are presented in **Supplementary Tables S3**, **S4**.

### 2.3 Prediction model

Genomic prediction was carried out with the model


(3) $$y^{*}=1\beta_{0}+Z_{f}u_{f}+Z_{d}u_{d}+e=1\beta_{0}+\left(Z_{f}\;Z_{d}\right)\left(\begin{array}{c} u_{f}\\ u_{d} \end{array}\right)+e$$

where *y* ^*^ is the vector of adjusted plot means, *β*_0_ is the intercept, *Z*_*f*_ and *Z*_*d*_ are the design matrices linking the marker effects to genotypes and *u*_*f*_,*u*_*d*_ are the marker effects vectors for testcross performance for the Flint and Dent parental components, respectively, of which the elements are $N\left(0,\sigma_{\text{TC}}^{2}\right)$ distributed, and *e* is the vector of residuals. The marker effects were estimated with the ridge regression best linear unbiased prediction (RR-BLUP) method (Whittaker et al., 2000; Meuwissen et al., 2001) using the R package rrBLUP (Endelman, 2011).

We further investigated the methods Bayes A, Bayes B (Meuwissen et al., 2001), and Bayesian ridge regression (BRR, de los Campos et al., 2009) using the R package BGLR Perez and de los Campos (2014). The BGLR package uses a Gibbs sampler to draw samples from posterior density, which we have set up the to perform 10000 iterations. The first 1000 iterations were used as burn-in. In the main part of this article we focus on the RR-BLUP method, a figure comparing the results obtained for complete set of methods is presented in the supplementary material (**Supplementary Figure S5**).

### 2.4 Training set designs

We used a re-sampling procedure to investigate different training set designs. In a first step, a random sample of 80 hybrids from each environment was drawn. We call these 80 hybrids the complete set for a certain environment. Each complete set was then divided randomly in two parts: a set called training set of size 60 and a set called prediction set of size 20. The complete set, training set, and prediction set were used according to the subsequently described designs for genomic prediction. The correlation between the predicted and observed performance of the hybrids was used as a measure for the prediction accuracy. To avoid stratification effects, correlations were computed independently at each environment using the Pearson’s correlation. For each investigated design the prediction was replicated for 100 cross validation runs. In each replication, the assignment of hybrids to the training set and the prediction set was the same for all designs.

**Figure 1:**
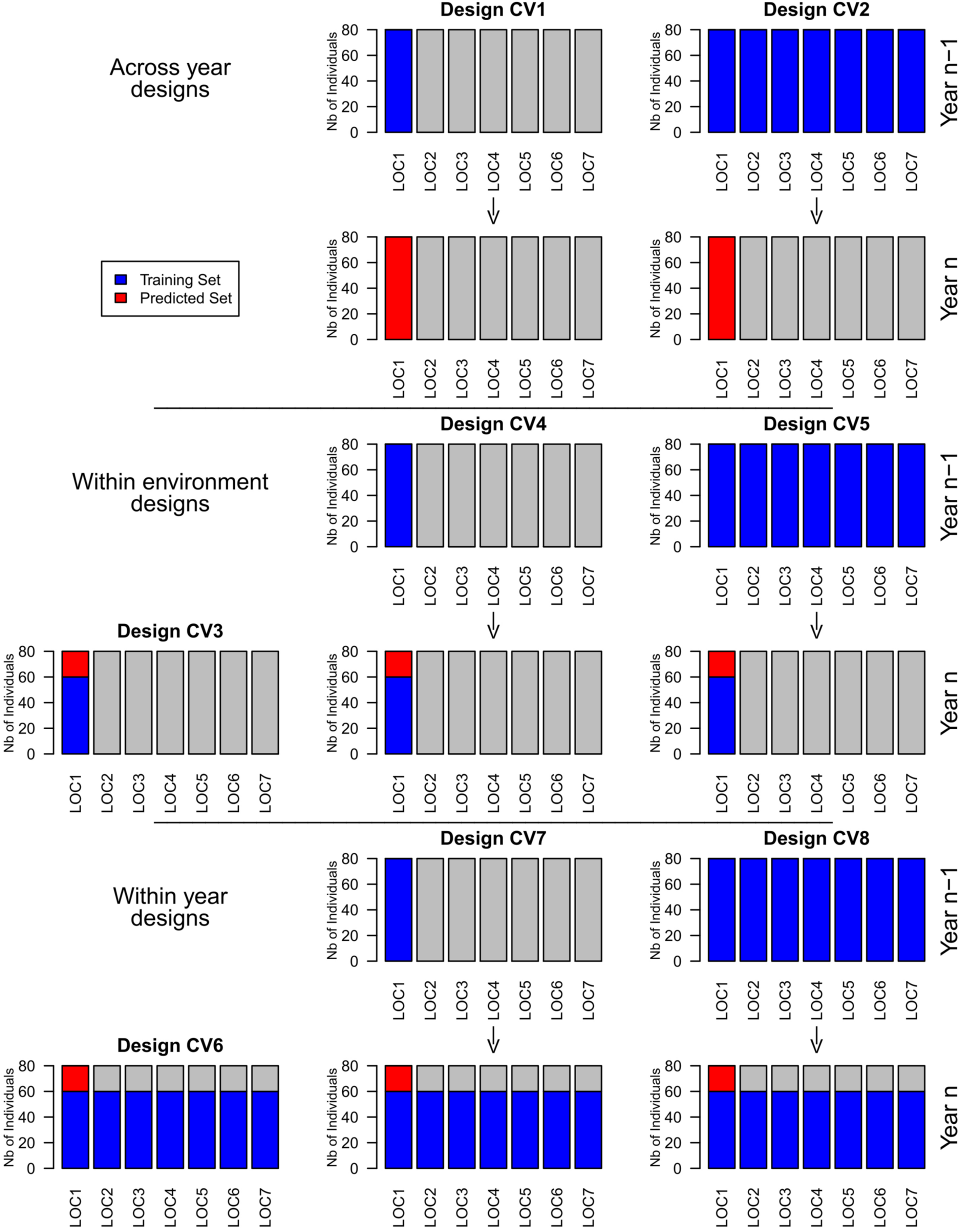
Schematic representation of the investigated training set designs for the prediction of location 1.

We investigated three groups of designs: Across year designs, within environment designs, and within year designs (**Figure 1**). The across year designs comprised designs CV1and CV2. In CV1, the design of a model consisted of the complete set of a certain location in one or two previous years. The model was used to predict the complete set of the respective location. In CV2, the design of a model consisted of all the complete sets from one or two previous years. The model was used to predict all complete sets of the current year.

The within environment designs comprised designs CV3, CV4, and CV5. In CV3, the design of a prediction model consisted of the training set of a certain environment. The model was used to predict the prediction set of the respective environment. CV4consisted of the design of CV3completed by the complete set of the same location in one or two previous years. The model was used to predict the prediction set of the respective location. CV5consisted of the design of CV3completed by the complete sets from one or two previous years. This model was used to predict the prediction set of the current year.

The within year designs comprised designs CV6, CV7, and CV8. In CV6, the design of a model consisted of all training sets from one year. The model was used to predict the prediction sets of the respective year. CV7consisted of the design of CV6completed by the complete set of a certain location in one or two previous years. The model was used to predict the prediction set of the same location in the current year. CV8consisted of the design of CV6completed by the complete sets from one or two previous years. These models were used to predict the prediction sets of the current year.

## 3 Results

**Figure 2:**
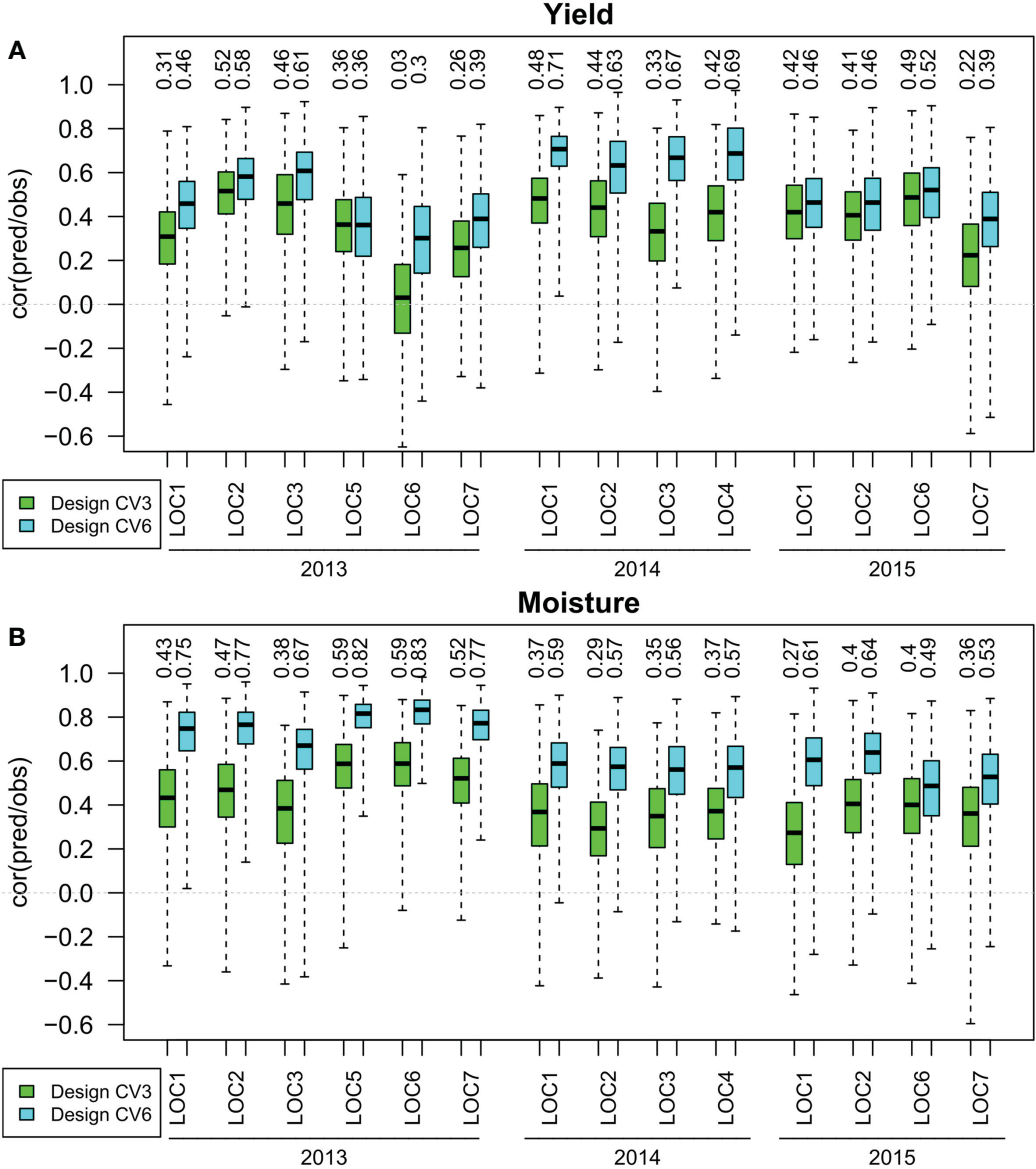
Correlation between observed and predicted yield **(A)** and moisture **(B)** for training set designs CV3(within environment with no previous data) and CV6(within year with no previous data) in years 2013 to 2015 at locations 1 to 7. The median of 100 simulations for the prediction method RR-BLUP is shown on top of the respective boxplots.

The prediction accuracy, assessed by the median of the correlation between predicted and observed phenotypes obtained in 100 cross validation runs, reached for the training set design CV3(within environment with no previous data) values between 0.03 and 0.52 for yield and between 0.27 and 0.59 for moisture using the RR-BLUP prediction method (**Figure 2**). A prediction accuracy greater than 0.5 was reached for yield in one and for moisture in three out of 14 environments. With the training set design CV6(within year with no previous data), prediction accuracies between 0.30 and 0.71 were reached for yield and prediction accuracies between 0.49 and 0.83 were reached for moisture. In seven out of 14 environments for yield, and in 13 out of 14 for moisture prediction accuracies greater than 0.5 were reached. The training set design CV6reached higher prediction accuracies than the design CV3in all environments for both traits.

**Figure 3:**
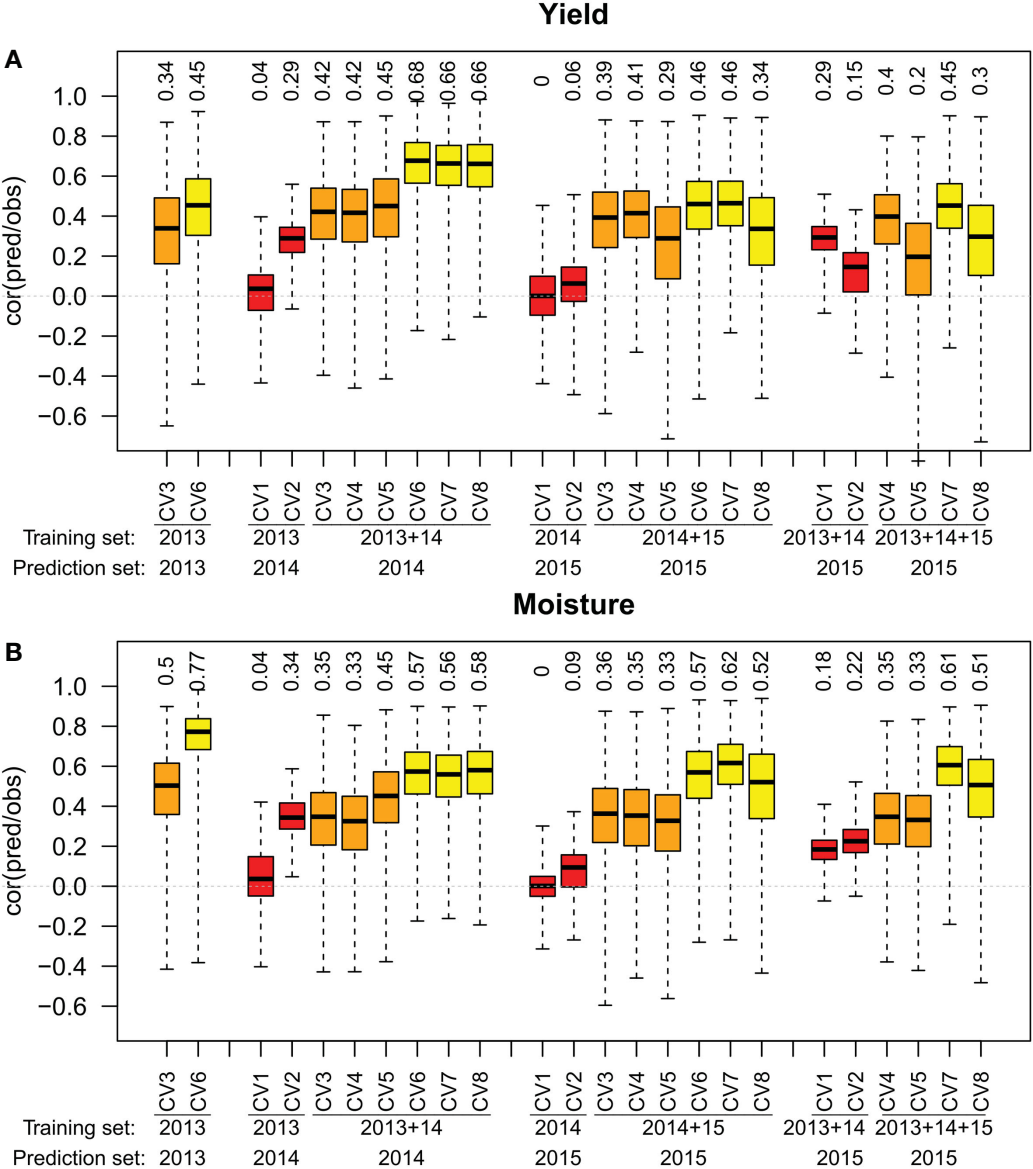
Correlation between observed and predicted yield **(A)** and moisture **(B)** for training set designs CV3(within environment with no previous data) and CV6(within year with no previous data) for one year, and training set designs CV1to CV8across one or two years. The median of 100 simulations for the prediction method RR-BLUP is shown on top of the respective boxplots.

When one previous year of data was used in the designs, prediction accuracies of the across year design CV1(across year with one previous location) ranged between 0 and 0.04 for both traits (**Figure 3**). Design CV2(across year with all previous locations) reached prediction accuracies between 0.06 and 0.29 for yield, and between 0.09 and 0.34 for moisture. The within environment designs CV3, CV4, and CV5reached prediction accuracies between 0.29 and 0.45 for yield and between 0.33 and 0.45 for moisture. The prediction accuracies of design CV4(within environment with one previous location) were slightly below those of design CV3(within environment with no previous data), except for yield in year 2015. The prediction accuracies of design CV5(within environment with all previous locations) were greater than those of design CV3in 2014 (+0.03 for yield and +0.10 for moisture) and lower in 2015 (-0.10 for yield and -0.03 for moisture). The within year designs CV6, CV7, and CV8reached prediction accuracies between 0.34 and 0.68 for yield and between 0.52 and 0.62 for moisture. Design CV7(within year with one previous location) reached greater prediction accuracies than CV6(within year with no previous data) for moisture in 2015 (+0.05). Design CV8(within year with all previous locations) reached greater prediction accuracies than design CV6only for moisture prediction in 2014 (+0.01). All within environment designs reached prediction accuracies greater than 0.5 except for yield in the year 2015. In all environments, within year designs outperformed within environment designs. Training set designs containing no genotypes from the predicted year were outperformed by both, within year and within environment designs.

When two previous years of data were available in year 2015, we compared the effect of using data from one or two previous years in the training set (**Figure 3**). Prediction accuracy of design CV1(across year with one previous location) increased by 0.29 for yield and by 0.18 for moisture when using data from 2013 in addition to data from 2014 in the training set. Prediction accuracy of design CV2(across year with all previous locations) increased by 0.09 for yield and by 0.13 for moisture. For design CV4(within environment with one previous location) the prediction accuracy decreased by 0.01 for yield and stayed the same for moisture. For design CV5(within environment with all previous locations), the prediction accuracies for moisture did not change, while prediction accuracies for yield decreased by 0.09. For design CV7(within year with one previous location), prediction accuracies decreased by 0.01 for both yield and moisture. For design CV8(within year with all previous locations), the prediction accuracies decreased by 0.04 for yield and by 0.01 for moisture. Across years designs benefited from the additional year while other designs remain unchanged or slightly decreased.

The results presented in **Figures 2**, **3** did not depend on the statistical prediction model. Similar results were observed for Bayes A, Bayes B, BRR and RR-BLUP effect estimation (**Supplementary Figure S5** for a design without previous year data and **Supplementary Figure S6** for a design with previous year data).

## 4 Discussion

### 4.1 Structure of the data set

The structure of our data set is characterized by the rather low number of 481 SNPs employed. These SNPs provided in previous cycles of the investigated breeding program prediction accuracies that were considered as sufficient by practical breeders (results not shown). This observation is supported by theoretical considerations. The lines used as parents of the investigated hybrids are related, as they are belonging to a heterotic pool of a hybrid breeding program. They consist of full-sibs (both crossing partners are the same) and half-sibs (one common crossing partner) derived from related crossing partners. Per definition, on a chromosome segment of 100 cM there occurs on average one crossing-over per meiosis. The maize chromosomes have lengths between 150 and 250 cM on most linkage maps. Consequently, common chromosome stretches that are shared by the hybrids are expected to be long and cover considerable parts of the chromosomes. For example, if a chromosome of length 200 cM consists of four long stretches in LD, then a marker distance of about 5 cM is expected to cover the recombination events on that chromosome with sufficient precision. Adding further markers is expected to increase the costs of genotyping without resulting in a substantial increase in the prediction accuracy. The average marker distances in our data set (**Supplementary Table S1**) and the LD decay plots (**Supplementary Figures S3**, **S4**) support these considerations. The average distance between two markers is around 5 to 6 cM on all chromosomes, and for marker distances around 5 cM strong LD is observed, with *r* ^2^ values around 0.3. We conclude that for the presented investigation the linkage map is sufficiently dense for precise genomic predictions.

The Roger’s distance is a measurement of the genetic distance between two populations based on the SNP composition of the individuals. It indicates how genetically close two populations are from each other and is related to heterosis and therefore often used in studies on hybrid breeding (Reif et al., 2005). On average, the pairwise Roger’s distance between hybrids in one location is between 0.125 and 0.140 except for the two locations 6 and 7 in 2015 (**Supplementary Table S2**). These two environments have the specificity to contain hybrids from two different testers (**Table 1**) which explain the highest average distance. The highest average pairwise Roger’s distance between the hybrids of two environments is 0.388. This is roughly twice the Roger’s distance observed within an environment. The reason is that in one location, hybrids share the same tester while when two locations are considered, two testers are involved. This statement is sustained by the average distance within one location for locations 6 and 7 in 2015. In these locations two testers have been used and the average distance is similar to the average distance of the hybrids in two locations.

The prediction accuracies observed for the CV6designs ranged mostly between 0.5 and 0.8 (**Figures 2**, **3**) and can be regarded as high and suitable for genomic selection of breeding material. A putative reason for the high prediction accuracy is that the lines are derived as doubled haploids from related crosses, resulting in rather long chromosome stretches which are identical by descent that can be marked by few genetic markers. Similar conclusions were drawn by Hickey et al. (2014). The unreplicated trial allows to test a large number of selection candidates, resulting in a greater selection intensity than that of replicated trials.

We conclude that unreplicated tests of small training sets and genotyping with a rather low number of markers allowed the screening of large numbers of selection candidates in our data set from early stages of a maize breeding program. Compared to phenotyping with replicated tests, the combination of unreplicated tests with genomic prediction seems to be a promising strategy for increasing the selection intensity.

### 4.2 Model used for estimating adjusted treatment values

In genomic prediction with replicated trials the phenotypic values are usually adjusted with a linear model. The resulting BLUPs of the genotypes (when regarding genotype as a random factor) or the least square means (LSMeans, for models with genotype as fixed factor) are used for the subsequent genomic prediction. Such a procedure is not possible for unreplicated trials, because if each genotype is evaluated only in one plot, the genotypic effect cannot be separated from the error term, and neither BLUPs nor LSMeans can be estimated. We therefore estimated the effects of year and environment within year with Equation 1 using standard analysis of variance methodology, and then adjusted the unreplicated phenotypes for each hybrid with the effects of years and environments. This is a common procedure in evaluating unreplicated trials.

### 4.3 Model used for genomic prediction

The statistical model in Equation 3 is a marker-based model that estimates testcross effects. Testcross effects cover additive gene effects and in addition a part of the dominance gene effects. The degree to which dominance effects are covered depends on the genetic separation of the two breeding pools. If the allele frequencies in the pools are differing to a large extent, then large portions of the dominance effects are included in the testcross effects. The fact that testcross effects include dominance is the backbone of hybrid breeding, and the only reason why hybrid breeding programs that employ testcrosses in early stages can successful realize selection gain. Our data set is from an ongoing commercial hybrid breeding program that employs testcrosses in early stages and the two pools are genetically separated (**Supplementary Figure S1**). Hence, in our dataset testcrosses are expected to cover a large part of the dominant gene action.

In experiments where all pairwise crosses of two sets of lines are carried out, the main effects of the lines are called general combining ability (GCA) effects and the interaction effects between fathers and mothers are called specific combining ability (SCA) effects. The GCA and SCA effects of such a factorial can be predicted with so called ‘animal models’ where the marker information is used to establish genomic relationship matrices (VanRaden, 2008), an example for the method is presented by [29]. Our data set consists of testcrosses of sets of candidate lines with inbred testers, in most instances a tested line is crossed to one tester only. This results in a confounding of GCA and SCA effects in the testcross effects. However, the SCA effects in ongoing hybrid breeding programs in maize are typically small (cf Schrag et al., 2009) and for this reason testcross values are used in many breeding programs as an approximation of the GCA values. Hence, including a SCA component in our model is not possible due to the structure of our experimental design. However, even if it were possible to add SCA effects to the model, an increase in prediction accuracy is not expected with the plant material of the investigated breeding program.

Interaction effects between SNPs and environment were not included in our genomic prediction model. Omitting the correction for environmental effects (Equation 1) and including environmental effects on the individual level and genotype by marker interaction effects in the genomic prediction model could accomplish this. Such an analysis would require that all different chromosome stretches in linkage disequilibrium that are occurring in the plant material are tested in a comparable replication number in all environments. Only then the unconfounded estimation of marker effects, environmental effects and marker × environment interaction would be possible. Such an analysis would need a planned balanced experiment, specifically designed to detect marker × environment interactions. In our data set such a balancedness is not guaranteed, neither for the tested lines nor for the testers. Employing a model as described above to our data would result in a strong confounding of genetic, environmental and interaction effects. Due to the rather high environmental effects this could result in severe under- or overestimation of the performance of the hybrids. We therefore decided to employ a correction for environmental effects (Equation 1) and to not include environmental effects and their interaction with genomic effects in the model.

### 4.4 Interaction between training set design and prediction method

The effect of the prediction model on the accuracy of genome wide prediction was investigated in numerous studies (e.g., Piepho, 2009; Li et al., 2020; Rolling et al., 2020) but without clear conclusions on the general preferability of a certain model. Comparing prediction models is not an objective of our paper, but we nevertheless wanted to check whether there is an interaction between the training set design and the employed prediction model. To accomplish this, we combined all training set designs with the prediction models Bayes A, Bayes B, BRR, and RR-BLUP and evaluated the prediction of yield and moisture in 2014 and 2015. To a large extent no differences in prediction accuracy were observed. Results for the design CV6are presented in the supplementary material (**Supplementary Figure S5**). Similar results were observed for all other training set designs (results not shown). Therefore we conclude that we have not detected an interaction between training set design and statistical model. We decided to use the RR-BLUP method for our investigations, as it is conceptually and computationally simpler than the other Bayes methods and it can be implemented using the fast and numerically stable R package rrBLUP.

### 4.5 Data used for model training

Test cross trials for the two heterotic pools of a hybrid breeding program are typically carried out in parallel. For example, in 2015 we tested at each of locations 1 and 2 the number of 106 Flint lines with a Dent tester. At locations 6 and 7 a total of 206 Dent lines were tested with four Flint testers (**Table 1**). Such data structures do allow two options for the tester side of the prediction model (Eq. 3). The first is to restrict the prediction model to the data points from a certain tester/test cross candidate pattern, i.e., to predict experiments with a pattern Flint tester/Dent test cross candidates, only with data from trials with this pattern. The second is to use in addition data points from the opposite tester/test cross pattern, i.e., to predict experiments with a pattern Flint tester/Dent test cross candidates, with experiments of both patterns, Flint tester/Dent test cross candidates, and Dent tester/Flint test cross candidates. We used the CV6training set design in years 2013 and 2015 to compare these alternatives and observed only marginal differences in prediction accuracy (results not shown). A study from Lorenzi et al., 2022 shows that both approaches give mostly similar results for sillage in maize. While these results does not point to obvious advantages of using both testing patterns in a training set, the larger amount of observations is expected to increase stability of numerical approaches and precision in small data sets as suggested by Technow et al. (2013). Therefore, we used both tester/testcross patterns for all training set designs involving more than one experiment.

### 4.6 Within year vs. within environment prediction

In a stand-alone one year test, the training set designs CV3and CV6are the only options that can be employed. From a quantitative genetics point of view, these designs differ in the genetic meaning of the estimated marker effects. In the design CV3, the marker effects are estimating the genotypic effect plus the genotype by environment interaction effect. In the design CV6, the effects estimate the genotypic effect only (neglecting for both cases the factor year and its interactions). While in replicated trials, estimating the genetic effect only is expected to provide the more precise estimation of genotypes, the situation in unreplicated trials is different. Each hybrid is tested only in one location, and therefore including the appropriate genotype by environment effect in the marker estimate might result in more precise prediction of the phenotype than the genetic effect alone. From a statistical point of view, the greater size of the training set for the CV6design is expected to result in more precise estimates of the genetic effects, which is expected to result in a more precise prediction of the phenotypes ([13]). These considerations were the motivation to compare the prediction accuracy of the CV6design with that of the CV3design. The CV6training set design resulted for all environments investigated in greater prediction accuracy than the CV3training set design (**Figure 2**).

We conclude that the greater precision of the effects estimated with the CV6training set design, consisting of several hundreds of hybrids, outweighs a putative positive effect from inclusion of the genotype by environment interaction in the CV3design. The CV6training set design delivers a sufficient prediction accuracy to be considered as an effective means to increase the selection gain in an unreplicated test cross trial.

### 4.7 Across year prediction

Across year prediction using training data from previous years is, under the condition of a sufficient prediction accuracy, an efficient method for pre-selection of promising candidates before testing them in the field (Wang et al., 2020). In the investigated data set, the training set designs CV1and CV2that employ across year prediction (**Figure 1**), resulted in the lowest prediction accuracies among the investigated training set designs (**Figure 3**). In across year predictions, environmental conditions of tested sets in two years differ in the year effect and genotype by environment interactions, while the phenotypic values from one year share the same year effect and genotype by environment interactions. The combination of both factors might serve as an explanation for the low prediction accuracy observed for the training set designs employing across year prediction. Our prediction accuracies are lower than those reached by (Wang et al., 2020) using only one previous year of data with multi-environment trials. However, as we use unreplicated trials, a lower prediction accuracy is expected. We found in barley (Terraillon et al., 2022) that even if prediction accuracy is low, across year genomic prediction in unreplicated trials might still be correlated to true genotypic values and therefore useful in selection despite genotype by environment effects. However this study is limited to a simulated barley dataset and the results do not necessarily apply to maize.

We conclude that with a data structure similar to our investigated experiment, the prediction accuracy reached with across year prediction is not sufficient for effective pre-selection of promising hybrids.

### 4.8 Adding data from one previous year to the within environment and within year designs

The training set designs CV3and CV6can be extended by including into the training set data from one previous year, either from the same location (training set designs CV4and CV7, **Figure 1**), or by including data from all locations used in previous year (training set designs CV5and CV8). Considering design CV3as a reference, both derived training set designs were not able to consistently outperform the reference (**Figure 3**). We observe that only in three cases over 12, the prediction accuracy is improved when we use previous years data. Interestingly, using all locations in 2014 improved the prediction accuracy for both yield and moisture in 2014 but it decreased it in 2015.

Considering CV6, the design CV8almost never reach a better prediction accuracies than CV6prediction (**Figure 3**). For the year 2015 it even resulted in a lower prediction accuracies for yield than CV3prediction. The design CV7reached in all investigated scenarios either a comparable or a slightly better prediction accuracy than the CV6design (**Figure 3**). However this increase is not consistent enough to recommand CV7over CV6.

We observed different situations when choosing for adding data from previous years to the training set. The prediction accuracies for moisture are especially illustrative. Considering moisture in 2014, for both, within environment as well as within year designs, adding data from previous years at the same location (Designs CV4and CV7) decreases the prediction accuracy compared to the initial training set design, while adding data from all locations (Designs CV5and CV8) increased it. For moisture in 2015, the situation is the opposite. For CV6based designs, adding same location increases the prediction accuracy while adding all locations decreases it. This increase can be explained by a more precise estimation of the phenotypes due to a more precise estimation of the marker by location effects. These results indicate that prediction accuracy in unreplicated trials is highly influenced by the year/environmental effects when considering across year predictions. Depending on the importance of the genotype by location effects in the considered years, adding one or all locations could decrease instead of increase prediction accuracy. Therefore there is a need to define in which conditions each approach should be privileged. Auinger et al. (2016) proposed to correct environmental/year effects using the same checks across years and managed to obtain good prediction accuracy with this method. Atanda et al. (2022) proposed sparse testing combined with overlap of tested material to achieve optimal prediction accuracy. These results were obtained in replicated trials however, we would expect similar results in unreplicated trials. Unfortunately, our experimental design did not include checks or overlapping material to test these hypothesis.

From an application point of view, adding data from previous years to the training sets of designs CV3or CV6seems not to be advantageous due to the variability of its effect, which might increase or decrease the prediction accuracy depending on the situation.

### 4.9 Adding data from two previous years to the training set

Extending the training set designs to incorporate two years of previous data instead of one (**Figure 3**) resulted in no increase of the prediction accuracy for the within environment and within year designs. For the across year designs CV1and CV2it resulted in an improvement of the prediction accuracy.

When the environmental effects of the predicted year are not available in the training population, then using multiple years of previous data could have a similar effect than using multiple locations. A similar result was observed by Wang et al. (2020). However, the prediction accuracy remains lower than for within environment and within year designs, as the genotype by environment effect cannot be assessed. It is expected, that increasing the number of years from which data is used in the training set can result in a further improvement of the prediction accuracy (Auinger et al., 2016). However, this applies only to situations where the genetic material is sufficiently related across years. In commercial breeding programs, this condition is often fulfilled, as selection candidates are commonly derived from related parents.

The lack of improvement in precision for within environment and within year designs could be explained by the major contribution of the data from the current year to a precise estimation of genome by environment effect. In comparison to this major contribution, earlier years can only contribute marginally to the prediction accuracy.

### 4.10 Impact of the training population size

The population size is often seen as one of the main components contributing to a high prediction accuracy (Liu et al., 2018; Sarinelli et al., 2019; Jarquin et al., 2020). However, in our study it had only a limited effect on the results.

Designs CV4and CV5compared to design CV3, and designs CV7and CV8compared to design CV6, have a much bigger population sizes (80 genotypes more for CV4and CV7and between 320 and 480 more for CV5and CV8) but show similar prediction accuracies. This result is confirmed when we consider prediction with data from two previous years. Here the number of extra genotypes is doubled while the prediction accuracy remains constant. Adding more genotypes from previous years to the training set of Design CV3or CV6do not increase the prediction accuracy, in most cases it even slightly decreases it.

These results suggest that for a precise prediction of unreplicated test crosses in maize a high grade of relatedness among the selection candidates and similar environmental conditions are more important than the pure number of genotypes in the training set. Including data from loosely related material evaluated in environments with different genotype by environment to the training set designs might even result in a negative effect on the prediction accuracy.

## Data availability statement

The data analyzed in this study is subject to the following licenses/restrictions: The data set is available on request from the authors. Requests to access these datasets should be directed to biometry.popgen@uni-giessen.de.

## Author contributions

MF conceived the study in collaboration with FR and CF. Analyses were performed by JT. The manuscript was written by JT and MF. FR and CF contributed to writing the manuscript. All authors contributed to the article and approved the submitted version.

## References

[B1] Albrecht T. Wimmer V. Auinger H.-J. Erbe M. Knaak C. Ouzunova M.. (2011). Genome-based prediction of testcross values in maize. Theor. Appl. Genet. 123, 339–350. doi: 10.1007/s00122-011-1587-7 21505832

[B2] Atanda S. A. Govindan V. Singh R. Robbins K. R. Crossa J. Bentley A. R. (2022). Sparse testing using genomic prediction improves selection for breeding targets in elite spring wheat. Theor. Appl. Genet. 135, 1939–1950. doi: 10.1007/s00122-022-04085-0 35348821 PMC9205816

[B3] Auinger H.-J. Schönleben M. Lehermeier C. Schmidt M. Korzun V. Geiger H. H.. (2016). Model training across multiple breeding cycles significantly improves genomic prediction accuracy in rye (Secale cereale l.). Theor. Appl. Genet. 129, 2043–2053. doi: 10.1007/s00122-016-2756-5 27480157 PMC5069347

[B4] Brauner P. C. Müller D. Molenaar W. S. Melchinger A. E. (2020). Genomic prediction with multiple biparental families. Theor. Appl. Genet. 133, 133–147. doi: 10.1007/s00122-019-03445-7 31595337

[B5] de los Campos G. Hickey J. M. Pong-Wong R. Daetwyler H. D. Calus M. P. L. (2013). Whole-genome regression and prediction methods applied to plant and animal breeding. Genetics 193, 327–345. doi: 10.1534/genetics.112.143313 22745228 PMC3567727

[B6] de los Campos G. Naya H. Gianola D. Crossa J. Legarra A. Manfredi E.. (2009). Predicting quantitative traits with regression models for dense molecular markers and pedigree. Genetics 182, 375–385. doi: 10.1534/genetics.109.101501 19293140 PMC2674834

[B7] Endelman J. B. (2011). Ridge regression and other kernels for genomic selection with r package rrblup. Plant Genome 4, 250–255. doi: 10.3835/plantgenome2011.08.0024

[B8] Endelman J. B. Atlin G. N. Beyene Y. Semagn K. Zhang X. Sorrells M. E.. (2014). Optimal design of preliminary yield trials with genome-wide markers. Crop Sci. 54, 48–59. doi: 10.2135/cropsci2013.03.0154

[B9] Fritsche-Neto R. Galli G. Borges K. L. R. Costa-Neto G. Alves F. C. Sabadin F.. (2021). Optimizing genomic-enabled prediction in small-scale maize hybrid breeding programs: A roadmap review. Front. Plant Sci. 12. doi: 10.3389/fpls.2021.658267 PMC8281958 34276721

[B10] Hickey J. M. Dreisigacker S. Crossa J. Hearne S. Babu R. Prasanna B. M.. (2014). Evaluation of genomic selection training population designs and genotyping strategies in plant breeding programs using simulation. Crop Sci. 54, 1476. doi: 10.2135/cropsci2013.03.0195

[B11] Jarquin D. Howard R. Crossa J. Beyene Y. Gowda M. Martini J. W. R.. (2020). Genomic prediction enhanced sparse testing for multi-environment trials. G3 Genes|Genomes|Genetics 10, 2725–2739. doi: 10.1534/g3.120.401349 32527748 PMC7407457

[B12] Li G. Dong Y. Zhao Y. Tian X. Würschum T. Xue J.. (2020). Genome-wide prediction in a hybrid maize population adapted to Northwest China. Crop J. 8, 830–842. doi: 10.1016/j.cj.2020.04.006

[B13] Liu X. Wang H. Wang H. Guo Z. Xu X. Liu J.. (2018). Factors affecting genomic selection revealed by empirical evidence in maize. Crop J. 6, 341–352. doi: 10.1016/j.cj.2018.03.005

[B14] Lorenz A. J. Smith K. P. (2015). Adding genetically distant individuals to training populations reduces genomic prediction accuracy in barley. Crop Sci. 55, 2657. doi: 10.2135/cropsci2014.12.0827

[B15] Lorenzi A. Bauland C. Mary-Huard T. Pin S. Palaffre C. Guillaume C.. (2022). Genomic prediction of hybrid performance: comparison of the efficiency of factorial and tester designs used as training sets in a multiparental connected reciprocal design for maize silage. Theor. Appl. Genet 135, 3143–3160. doi: 10.1007/s00122-022-04176-y 35918515

[B16] Massman J. M. Gordillo A. Lorenzana R. E. Bernardo R. (2013). Genomewide predictions from maize single-cross data. Theor. Appl. Genet. 126, 13–22. doi: 10.1007/s00122-012-1955-y 22886355

[B17] Meuwissen T. H. E. Hayes B. J. Goddard M. E. (2001). Prediction of total genetic value using genome-wide dense marker maps. Genetics 157, 1819–1829. doi: 10.1093/genetics/157.4.1819 11290733 PMC1461589

[B18] Michel S. Ametz C. Gungor H. Akgöl B. Epure D. Grausgruber H.. (2017). Genomic assisted selection for enhancing line breeding: merging genomic and phenotypic selection in winter wheat breeding programs with preliminary yield trials. Theor. Appl. Genet. 130, 363–376. doi: 10.1007/s00122-016-2818-8 27826661 PMC5263211

[B19] Michel S. Löschenberger F. Ametz C. Pachler B. Sparry E. Bürstmayr H. (2019). Simultaneous selection for grain yield and protein content in genomics-assisted wheat breeding. Theor. Appl. Genet. 132, 1745–1760. doi: 10.1007/s00122-019-03312-5 30810763 PMC6531418

[B20] Perez P. de los Campos G. (2014). Genome-wide regression and prediction with the bglr statistical package. Genetics 198, 483–495. doi: 10.1534/genetics.114.164442 25009151 PMC4196607

[B21] Piepho H. P. (2009). Ridge regression and extensions for genomewide selection in maize. Crop Sci. 49, 1165–1176. doi: 10.2135/cropsci2008.10.0595

[B22] Reif J. C. Melchinger A. E. Frisch M. (2005). Genetical and mathematical properties of similarity and dissimilarity coefficients applied in plant breeding and seed bank management. Crop Sci 45, 1–7. doi: 10.2135/cropsci2005.0001

[B23] Rincent R. Laloë D. Nicolas S. Altmann T. Brunel D. Revilla P.. (2012). Maximizing the reliability of genomic selection by optimizing the calibration set of reference individuals: Comparison of methods in two diverse groups of maize inbreds ( *Zea mays* l.). Genetics 192, 715–728. doi: 10.1534/genetics.112.141473 22865733 PMC3454892

[B24] Rolling W. R. Dorrance A. E. McHale L. K. (2020). Testing methods and statistical models of genomic prediction for quantitative disease resistance to phytophthora sojae in soybean [Glycine max (L.) merr] germplasm collections. Theor. Appl. Genet. 133, 3441–3454. doi: 10.1007/s00122-020-03679-w 32960288

[B25] Sarinelli J. M. Murphy J. P. Tyagi P. Holland J. B. Johnson J. W. Mergoum M.. (2019). Training population selection and use of fixed effects to optimize genomic predictions in a historical USA winter wheat panel. Theor. Appl. Genet. 132, 1247–1261. doi: 10.1007/s00122-019-03276-6 30680419 PMC6449317

[B26] Schrag T. A. Möhring J. Maurer H. P. Dhillon B. S. Melchinger A. E. Piepho H. P.. (2009). Molecular marker-based prediction of hybrid performance in maize using unbalanced data from multiple experiments with factorial crosses. Theor. Appl. Genet. 118, 741–751. doi: 10.1007/s00122-008-0934-9 19048224

[B27] Schrag T. A. Schipprack W. Melchinger A. E. (2019). Across-years prediction of hybrid performance in maize using genomics. Theor. Appl. Genet. 132, 933–946. doi: 10.1007/s00122-018-3249-5 30498894

[B28] Technow F. Bürger A. Melchinger A. E. (2013). Genomic prediction of northern corn leaf blight resistance in maize with combined or separated training sets for heterotic groups. G3 Genes|Genomes|Genetics 3, 197–203. doi: 10.1534/g3.112.004630 23390596 PMC3564980

[B29] Technow F. Riedelsheimer C. Schrag T. A. Melchinger A. E. (2012). Genomic prediction of hybrid performance in maize with models incorporating dominance and population specific marker effects. Theor. Appl. Genet. 125, 1181–1194. doi: 10.1007/s00122-012-1905-8 22733443

[B30] Terraillon J. Frisch M. Falke K. C. Jaiser H. Spiller M. Cselényi L.. (2022). Genomic prediction can provide precise estimates of the genotypic value of barley lines evaluated in unreplicated trials. Front. Plant Sci. 13. doi: 10.3389/fpls.2022.735256 PMC9072862 35528936

[B31] VanRaden P. (2008). Efficient methods to compute genomic predictions. J. Dairy Sci. 91, 4414–4423. doi: 10.3168/jds.2007-0980 18946147

[B32] Verges V. L. Van Sanford D. A. (2020). Genomic selection at preliminary yield trial stage: Training population design to predict untested lines. Agronomy 10, 60. doi: 10.3390/agronomy10010060

[B33] Wang N. Wang H. Zhang A. Liu Y. Yu D. Hao Z.. (2020). Genomic prediction across years in a maize doubled haploid breeding program to accelerate early-stage testcross testing. Theor. Appl. Genet. 133, 2869–2879. doi: 10.1007/s00122-020-03638-5 32607592 PMC7782462

[B34] Wang X. Xu Y. Hu Z. Xu C. (2018). Genomic selection methods for crop improvement: Current status and prospects. Crop J. 6, 330–340. doi: 10.1016/j.cj.2018.03.0018

[B35] Whittaker J. C. Thompson R. Denham M. C. (2000). Marker-assisted selection using ridge regression. Genet. Res. 75, 249–252. doi: 10.1017/S0016672399004462 10816982

