# Supplementary material for: Training set designs for prediction of yield and moisture of maize test cross hybrids with unreplicated trials
Source: Front Plant Sci. 2023 Mar 6;14:1080087. doi: 10.3389/fpls.2023.1080087 (PMC10025381; doi:10.3389/fpls.2023.1080087)
Supplement: Supplementary file 1 [file DataSheet_1.pdf]

Supplementary Material

SUPPLEMENTARY TABLES AND FIGURES

Table S1. Markers repartition over the chromosomes. The average distance between two markers and the maximum distance between two markers are based on the genetic distance in centiMorgan (cM).

Markers repartition over the chromosomes

Chromosome	Number of markers	Average distance (cM)	Maximum distance (cM)
1	72	3.078	16.34
2	57	3.233	18.03
3	67	2.845	30.75
4	44	3.624	28.01
5	68	2.541	14.27
6	30	5.375	20.84
7	49	2.998	26.62
8	40	3.660	20.09
9	26	4.662	24.44
10	28	4.393	31.25

Table S2. Average pairwise distance between hybrids in one or two environment(s). Pairwise distance is calculated using the Roger's distance (RD) between SNPs markers. An environment is defined as a year/location combination. The first line of the table shows average pairwise distance in one environment. The triangle matrix shows the average pairwise distance between two environments.

		Year13						Year14				Year15			
		Loc1	Loc2	Loc3	Loc5	Loc6	Loc7	Loc1	Loc2	Loc3	Loc4	Loc1	Loc2	Loc6	Loc7
Average pairwise distance between hybrids in one environment															
		0.140	0.138	0.135	0.138	0.140	0.137	0.137	0.132	0.133	0.132	0.125	0.135	0.217	0.232
Average pairwise distance between hybrids in two environments															
Year13	Loc1														
	Loc2	0.272													
	Loc3	0.351	0.297												
	Loc5	0.328	0.286	0.326											
	Loc6	0.285	0.244	0.283	0.294										
	Loc7	0.282	0.239	0.280	0.300	0.228									
Year14	Loc1	0.388	0.353	0.382	0.377	0.369	0.365								
	Loc2	0.339	0.306	0.335	0.317	0.332	0.335	0.358							
	Loc3	0.296	0.265	0.293	0.317	0.215	0.269	0.369	0.331						
	Loc4	0.272	0.240	0.270	0.279	0.222	0.227	0.357	0.318	0.254					
Year15	Loc1	0.266	0.253	0.310	0.296	0.253	0.251	0.367	0.318	0.279	0.248				
	Loc2	0.328	0.290	0.274	0.311	0.271	0.269	0.380	0.327	0.291	0.260	0.302			
	Loc6	0.313	0.280	0.301	0.331	0.250	0.295	0.385	0.313	0.185	0.283	0.298	0.305		
	Loc7	0.295	0.266	0.287	0.304	0.268	0.273	0.374	0.278	0.294	0.203	0.280	0.286	0.298	

Table S3. Year effects. Model used to calculate year effects is presented in the material and method of Terraillon et al, 2022.

Year effect

	2013	2014	2015
Yield	-0.618	1.096	-0.241
Moisture	1.740	-2.519	0.114

Table S4. Environment effects. Environment is defined as a Year/Location combination. Model used to calculate environmental effects is presented in the material and method of Terraillon et al, 2022.

Environment effect (Year:Location)

	Year13						Year14				Year15			
	Loc1	Loc2	Loc3	Loc5	Loc6	Loc7	Loc1	Loc2	Loc3	Loc4	Loc1	Loc2	Loc6	Loc7
Yield	0.08	0.04	0.87	-0.36	-0.73	0.14	0.35	-0.23	-0.28	0.15	-0.35	0.53	-0.31	0.12
Moisture	0.29	0.23	0.31	-0.01	-0.88	0.01	1.87	0.18	-1.07	-0.98	-0.61	0.28	0.27	0.05

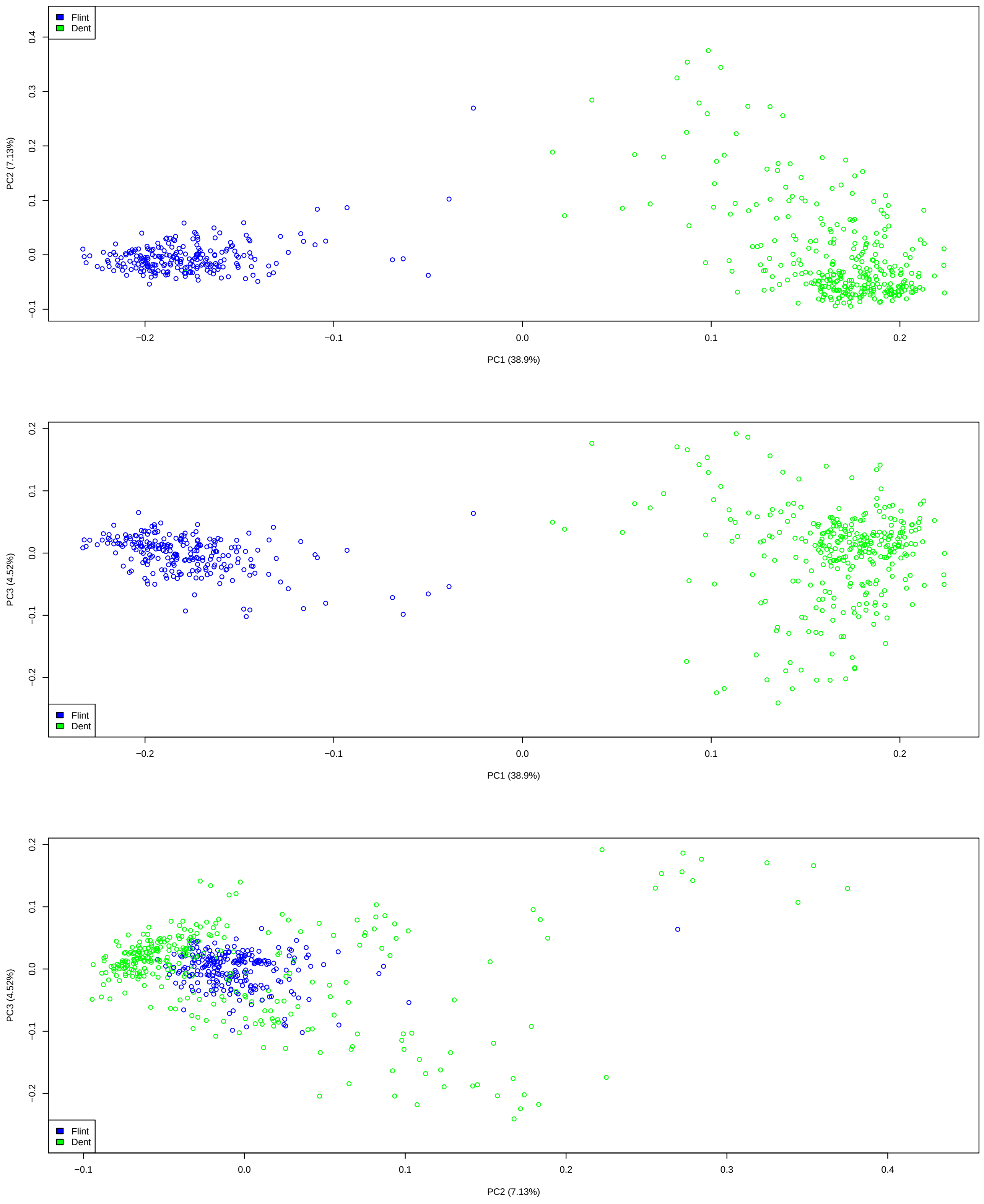

Figure S1. PCA (Principal Component Analysis) with 3 principal components based on SNP markers information for all lines. Percentage explained by principal components is indicated between brackets for each axis. Flint lines are presented in blue, Dent lines in green.

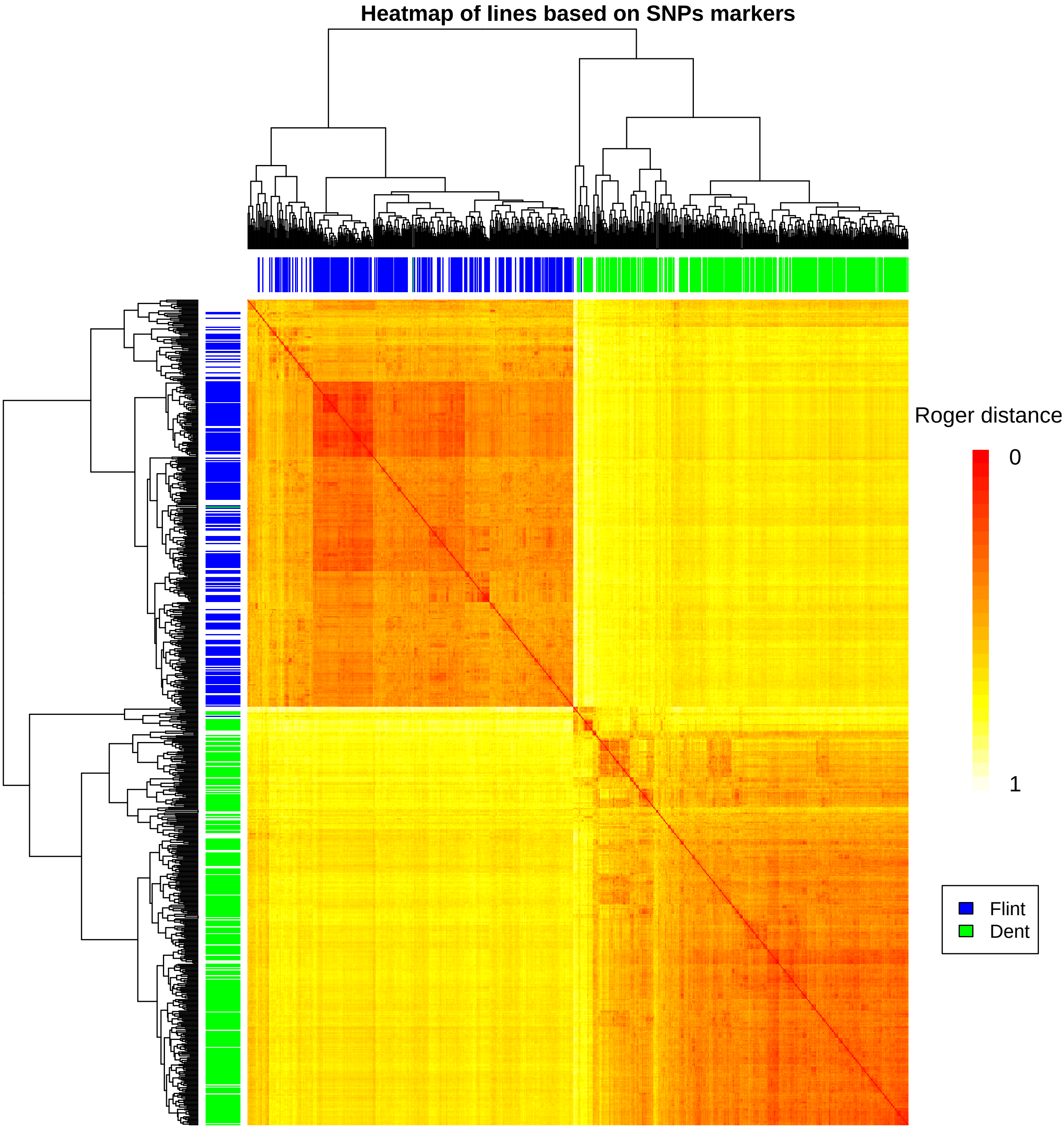

Figure S2. Heatmap of the lines based on the SNP markers information. Distance between lines is calculated using the Roger's distance. Flint lines are indicated in blue, Dent lines in green. Lines are ordered by a dendrogram on both X and Y axis.

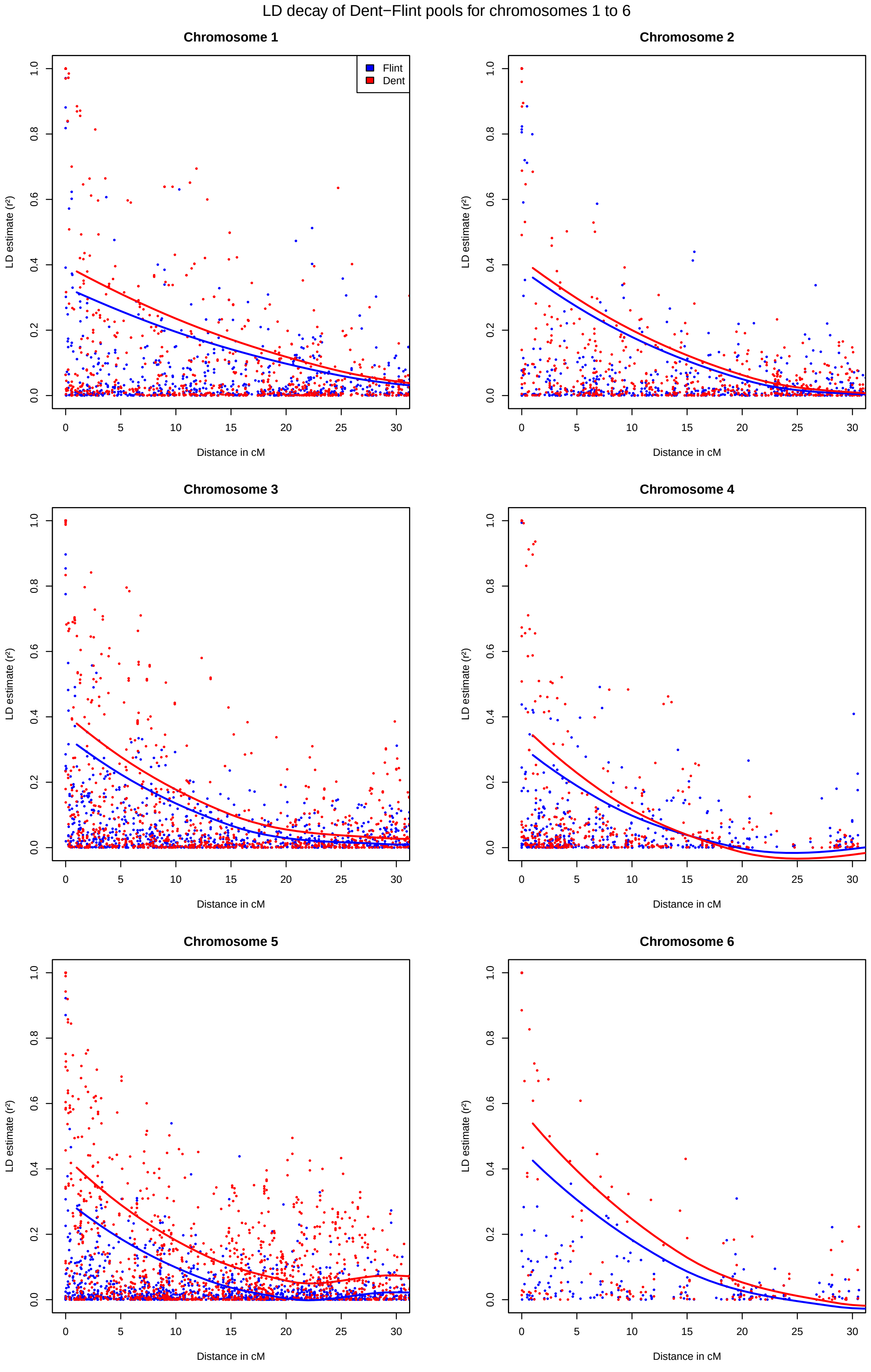

Figure S3. LD decay for Flint and Dent pools Part 1: Linkage disequilibrium decay for chromosomes 1 to 6 based on the genetic map information. LOESS regression lines are indicated in blue for the Flint pool, in red for the Dent pool.

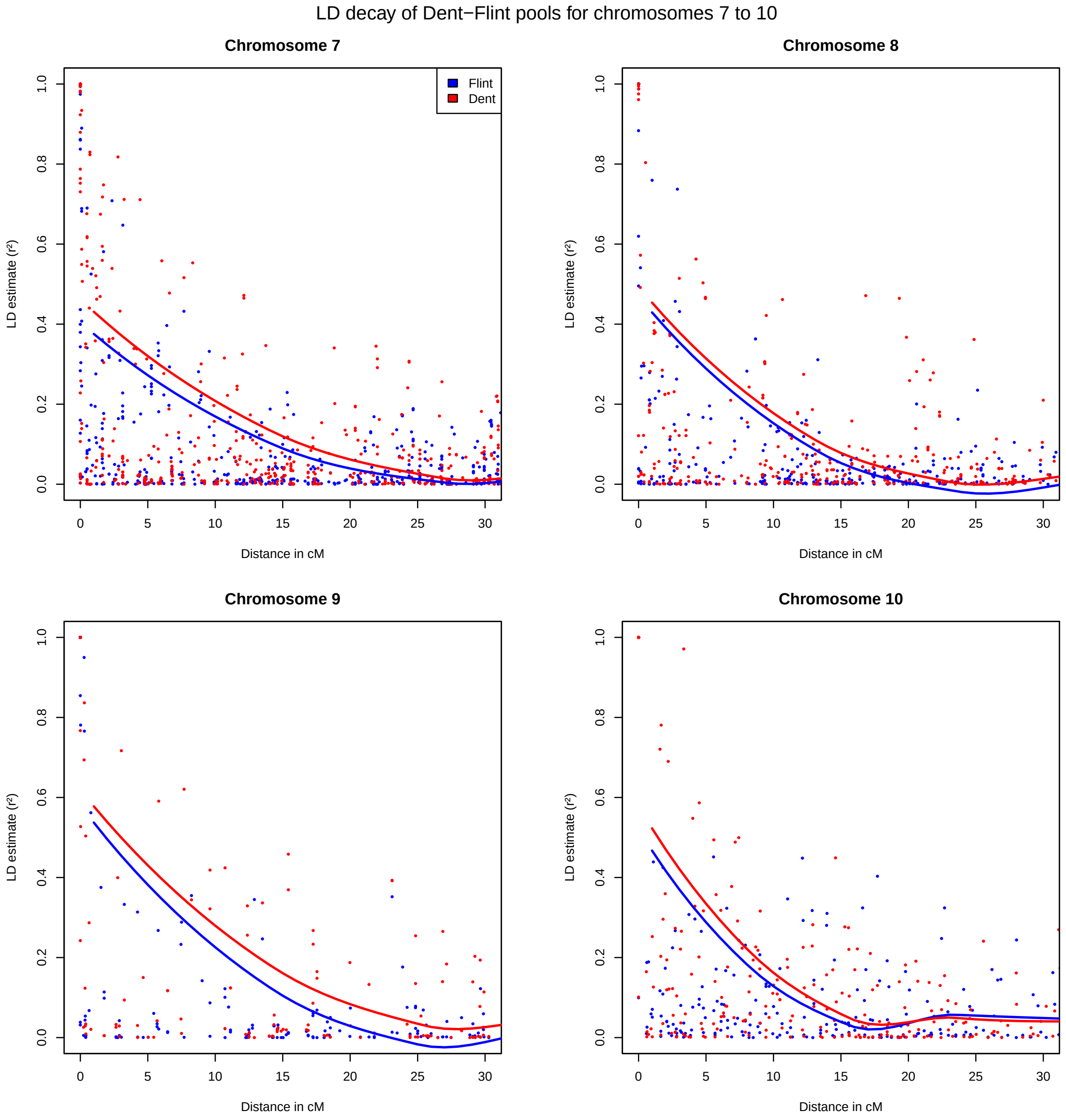

Figure S4. LD decay for Flint and Dent pools Part 2: Linkage disequilibrium decay for chromosomes 7 to 10 based on the genetic map information. LOESS regression lines are indicated in blue for the Flint pool, in red for the Dent pool.

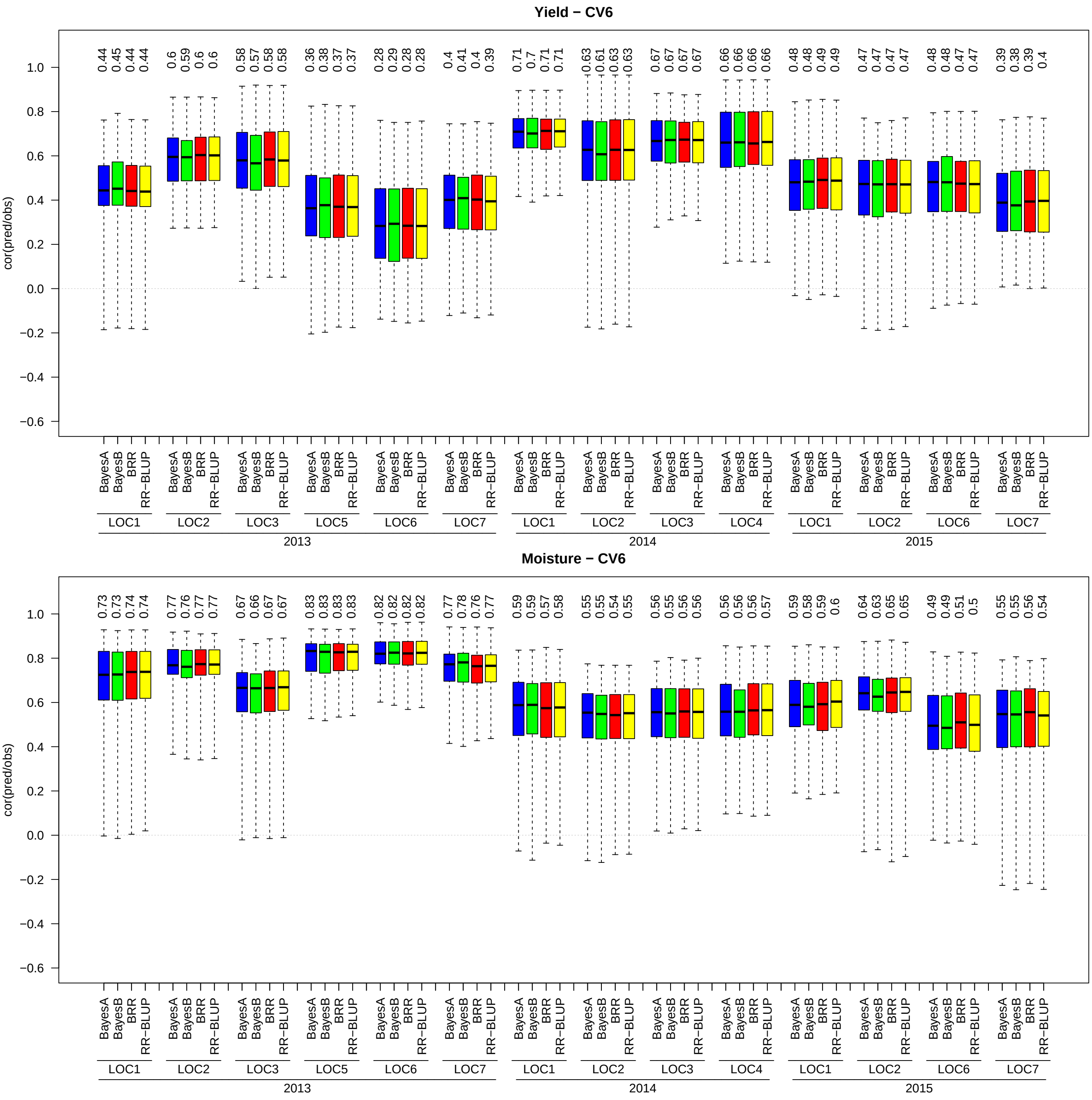

Figure S5. Correlation between observed and predicted yield and moisture for training set CV6 using four prediction methods: Bayes A, Bayes B, Bayesian ridge regression (BRR) and RR-BLUP. The median of 100 simulations for the predictions is shown on the top of the respective boxplots.

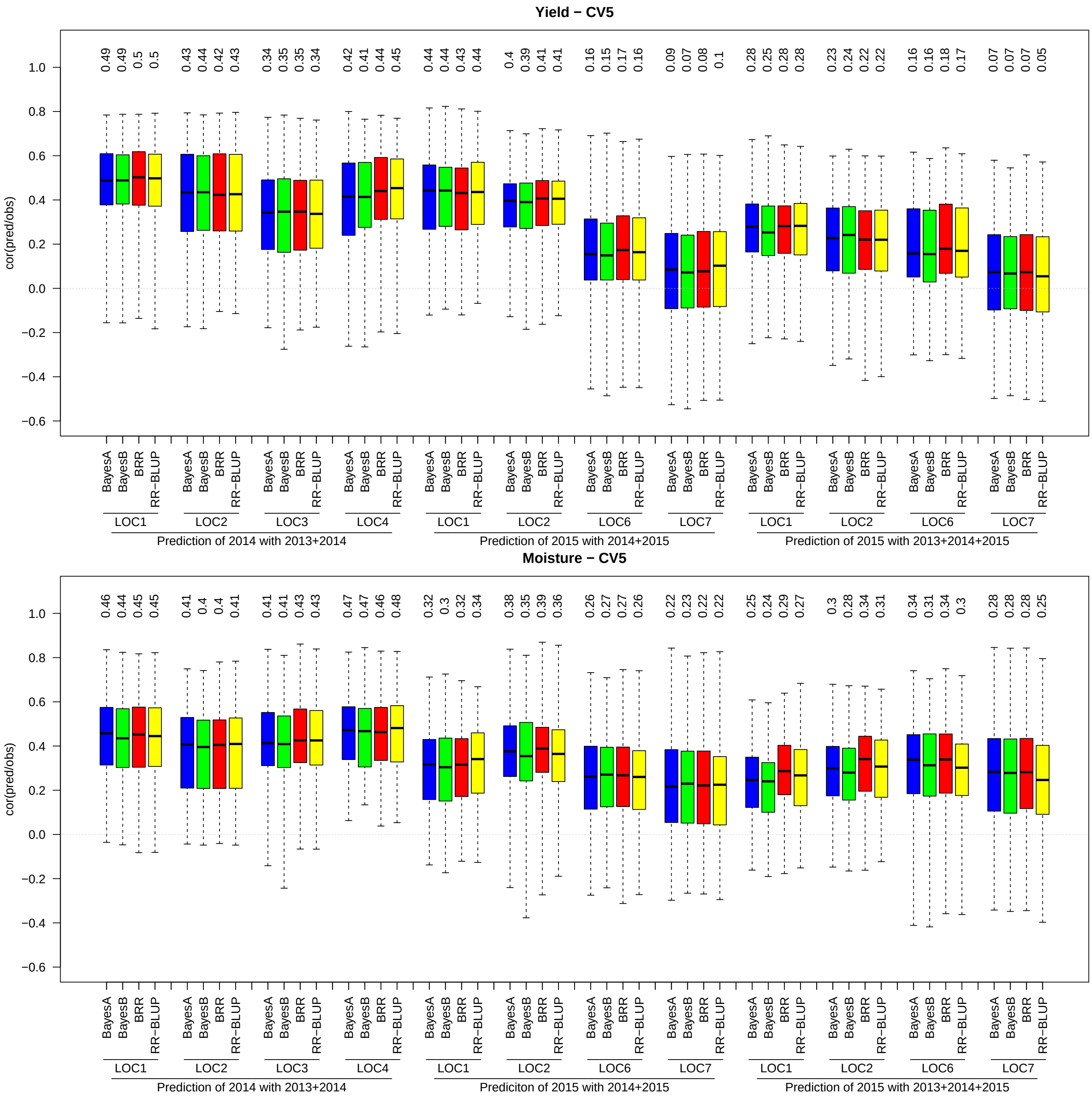

Figure S6. Correlation between observed and predicted yield and moisture for training set CV5 using four prediction methods: Bayes A, Bayes B, Bayesian ridge regression (BRR) and RR-BLUP. The median of 100 simulations for the predictions is shown on the top of the respective boxplots.
